# Enhancing melanoma treatment through systemic delivery of an immune boosting *Staphylococcus epidermidis* strain

**DOI:** 10.1038/s41598-025-20581-x

**Published:** 2025-10-21

**Authors:** Jeewon Hwang, Gwanghee Kim, Yoojin Lee, Mohammed Ali, Junho Cho, Sang Sun Yoon

**Affiliations:** 1 Department of Microbiology and Immunology, Yonsei University College of Medicine, 50-1 Yonsei-ro, Seodaemun-gu, Seoul, 03722 Republic of Korea; 2 Brain Korea 21 Project for Medical Sciences, Yonsei University College of Medicine, Seoul, Republic of Korea; 3 Institute of Immunology and Immunological Diseases, Yonsei University College of Medicine, Seoul, Republic of Korea; 4 BioMe Inc., Seoul, Republic of Korea

**Keywords:** *Staphylococcus epidermidis*, Melanoma, Immunotherapy, Immune checkpoint inhibitors, Skin microbiota, Anti-tumor, Cell biology, Immunology, Microbiology, Physiology, Oncology

## Abstract

A unique strain of *Staphylococcus epidermidis*, AIT01 (AIT, Airway Immune Trainer), identified in our previous research, has demonstrated immune-boosting properties. This study aimed to evaluate the systemic immune-modulatory effects and potential anti-tumor properties of this immune-enhancing skin microbiota strain. A series of ex vivo and in vivo experiments were conducted to assess immune cell proliferation, cytokine production, and anti-tumor efficacy. In ex vivo studies, splenocytes treated with the bacterial lysate or culture supernatant of the strain showed significantly increased viability in a concentration-dependent manner. Flow cytometry analysis revealed increased populations of dendritic cells, NK cells (Natural killer cells), and γδ T cells, with enhanced cytokine production, particularly IFN-γ (Interferon-γ) and perforin, in the lysate-treated group. When administered via intraperitoneal and intravenous routes in vivo, mice showed significant inhibition of melanoma growth upon receiving the bacterial lysate. Notably, pre-treatment demonstrated superior efficacy compared to post-treatment. Furthermore, the combination of the bacterial lysate with anti-PD-1 (anti-Programmed cell death protein-1) monoclonal antibody further suppressed tumor growth compared to anti-PD-1 monotherapy. These findings suggest that the AIT01 lysate enhances immune cell proliferation and cytokine production, contributing to its potent anti-tumor effects. The systemic delivery of this immune-boosting skin microbiota strain, particularly in combination with anti-PD-1 therapy, holds promise as an effective immunotherapeutic strategy against melanoma.

**Supplementary Information:**

The online version contains supplementary material available at 10.1038/s41598-025-20581-x.

## Introduction

The microbiota plays a crucial role in maintaining host health by interacting closely with various body parts, such as the skin, lungs, and intestines^1–3^. The gut microbiota, in particular, has attracted significant attention due to its extensive influence on the host’s immune system. Various studies have demonstrated that it enhances anti-tumor immune responses by inducing the accumulation of neutrophils, NK cells, CD4 + and CD8 + T cells, and the production of IFN-γ^4^. Furthermore, the gut microbiota has been found to be linked with a range of diseases, including heart disease, inflammatory bowel disease, hepatitis, asthma, diabetes, and cancers such as lung, colorectal, pancreatic, and oral cancers^5–7^.

Next-generation sequencing (NGS) has revealed low-biomass intratumor microbiota within various cancer tissues, indicating that these microbiota could affect tumorigenesis, cancer treatment resistance, and prognosis^8–10^. Co-treatment with *Lactobacillus rhamnosus* GG and anti-PD-1 antibodies has shown promising anti-tumor effects by activating CD8^+^ T cells through TLR2 (Toll-like receptor) recognition^11,12^. Immunotherapy, which utilizes the patient’s immune system to fight cancer, is one of the most promising approaches in current cancer treatment^13^. Immune checkpoint inhibitors, such as anti-PD-1 or anti-CTLA-4 (anti-Cytotoxic T-lymphocyte-associated protein 4) antibodies, have exhibited significant inhibitory effects on cancer growth^13,14^. Blocking CTLA-4 promotes T-cell activation during the immune response’s priming phase while inhibiting the PD-1 pathway restores tumor-specific T-cell functions, leading to cancer cell elimination and the production of immunostimulatory cytokines like IFN-γ, IL-2, and TNF-α^15,16^.

The interaction between gut microbiota and host immunity has received significant attention, but the immunological impact of skin microbiota is not as well understood^17–20^. Harnessing the skin microbiota to stimulate the host’s immune response against skin cancer could be a promising strategy, considering the critical role of the immune system in cancer treatment^21,22^. *Staphylococcus epidermidis*, a common bacterium found on the skin and in the nasal cavity^3^, has potential benefits for skin health^23^. It activates dendritic cells, aids in pathogen defense, supports T-cell accumulation, and assists in wound repair^24^. Moreover, it has been reported to inhibit melanoma without systemic toxicity through a substance called 6-HAP (6-N-hydroxyaminopurine)^25^.

Our previous research discovered a unique *S. epidermidis* isolate called AIT01 (AIT, Airway Immune Trainer). AIT01 exhibited pro-inflammatory effects by stimulating cytokine production and innate immune responses, resulting in notable anti-bacterial and anti-viral effects^23^. Interestingly, recent studies indicate that anti-infective factors can also aid in anticarcinogenic treatment^26^.

Accordingly, we investigated whether the systemic delivery of AIT01, with its immune-enhancing properties, could induce anti-tumor effects. We confirmed that AIT01 increased the viability of immune cells (splenocytes), the number of immune cells (dendritic cells, NK cells, and γδ T cells), and the production of cytokines (IFN-γ and perforin) in NK cells. Both intraperitoneal and intravenous administration of AIT01 lysate displayed tumor-suppressive effects in a melanoma animal model. Additionally, the combined treatment of aPD-1 and AIT01 significantly inhibited melanoma growth.

## Results

### AIT01 increases the viability of various immune cells

AIT01 has been previously shown to enhance the viability of various immune cells and stimulate non-specific airway immune responses, contributing to antiviral defense^23^. This study aimed to determine whether AIT01 could also trigger systemic immunity in cancer by evaluating its impact on immune cells and cancer cells.

We initially assessed the viability of immune cells with AIT01 by examining cell proliferation and cytotoxicity. The spleen was selected as the source of immune cells because it contains a variety of immune cell populations, and an ample number of cells compared to lymph nodes. We studied two different ways of administering AIT01: bacterial lysate and bacterial culture supernatant (CS). We didn’t use live bacteria due to safety concerns, especially the risk of sepsis.

**Fig. 1:**
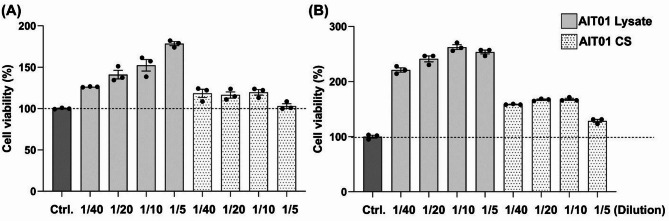
AIT01 enhances the viability of various immune cells. Cell viability at OD_600_ after treating splenocytes with AIT01 lysate and supernatant for (**A**) 6 h or (**B**) 24 h. The results were standardized by the viability value of the control.

To estimate the viability of splenocytes with AIT01 lysate or CS, we utilized the cell counting kit-8 (CCK-8) assay. After 6 h of co-culture, the immune cell viability increased in response to the treatment with AIT01 lysate in a concentration-dependent manner. The highest viability was observed at the lowest dilution factor (1:5), with a value of 178.34%. As the dilution factor increased, the viability decreased progressively, showing values of 152.28% at 1:10, 140.95% at 1:20, and 126.23% at 1:40 (Fig. 1A). In contrast, the supernatant treatment did not show a clear dilution-dependent pattern, though it also increased cell viability compared to the control (values ranging between 103.05% and 119.63%) (Fig. 1A). After 24 h of co-culture, the immune cell viability for AIT01 lysate treatment increased significantly across all dilution factors, again showing a dilution-dependent trend (253.57%, 262.50%, 241.07%, and 220.98% at 1:5, 1:10, 1:20, and 1:40 dilutions, respectively) (Fig. 1B).

In conclusion, both AIT01 lysate and supernatant treatments resulted in increased immune cell viability compared to the control group. Notably, the lysate treatment exhibited more than double the immune cell viability compared to the control group, indicating a higher efficacy in immune cell proliferation. These findings suggest that both AIT01 lysate and supernatant promote immune cell proliferation, with the lysate being particularly effective.

### AIT01 increases the population of immune cells and stimulates the production of cytokines

We confirmed that AIT01 improved the viability of immune cells. The lysate showed better results than the culture supernatant (CS). However, it was unclear whether the improved cell viability with AIT01 was associated with anti-tumor effects. Therefore, we conducted a flow cytometry analysis to investigate specific changes in immune cells in splenocytes with AIT01 treatment.

**Fig. 2:**
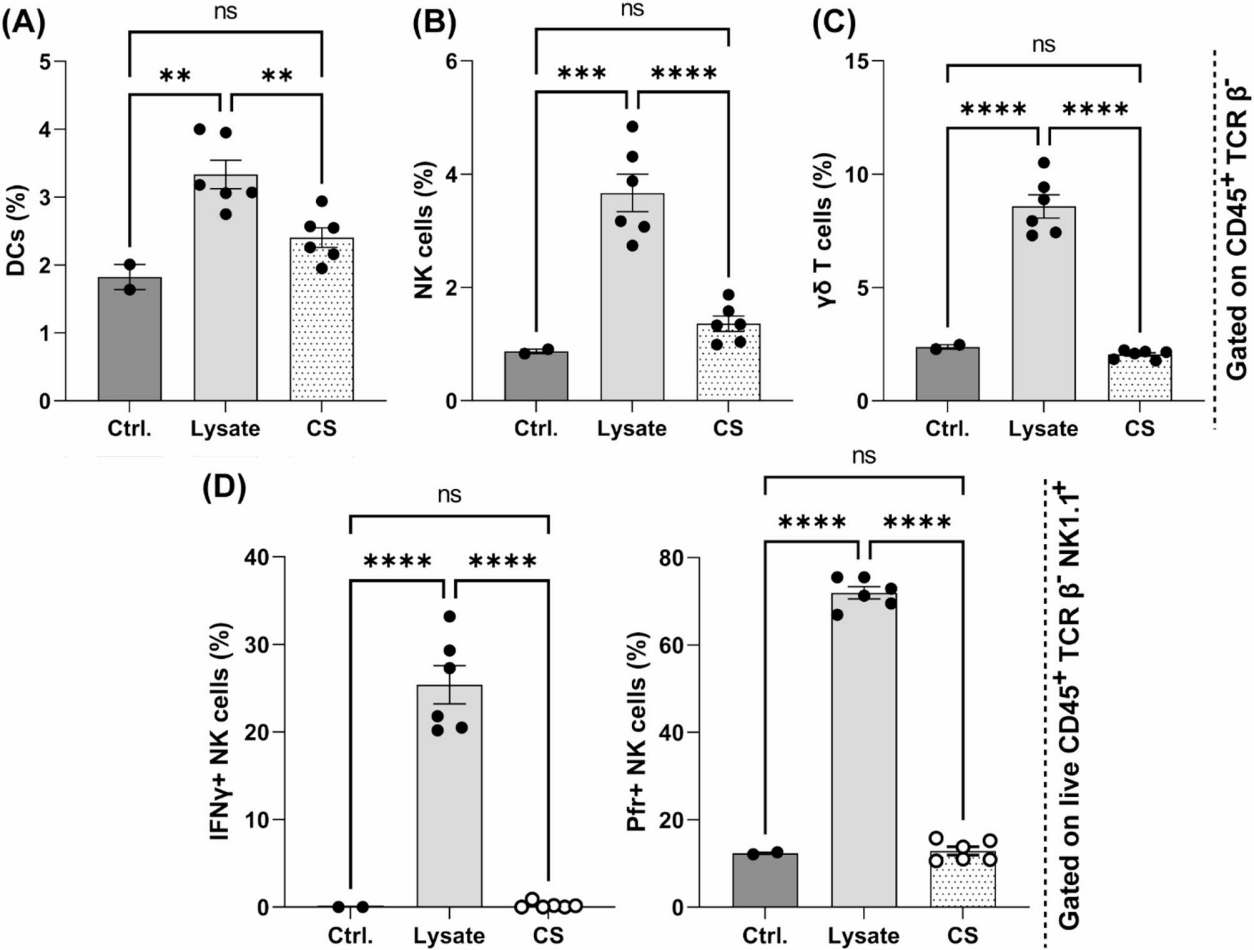
AIT01 increases the population of immune cells and stimulates their cytokine production ability for anti-tumor effects. (**A**–**C**) Treatment of splenocytes with AIT01 lysate and supernatant resulted in significant changes in immune cell populations. The immune cells were identified by gating for CD45^+^ / TCR β^−^ using flow cytometry analysis. (**D**) The changes in NK cells’ cytokine production ability for anti-tumor effects. The flow cytometry analysis of cytokine production in NK cells was conducted with CD45^+^ / TCR β^−^ / NK1.1^+^ gating. “Ctrl.” refers to the control, “Lysate” refers to AIT01 lysate, and “CS” refers to AIT01 culture supernatant. The asterisks indicate: * for a p-value of 0.05, ** for a p-value of ≤ 0.01, *** for a p-value of ≤ 0.001, and **** for a p-value of ≤ 0.0001.

The treatment of AIT01 lysate significantly increased the population of dendritic cells (DCs), which are potent antigen-presenting cells that stimulate tumor-specific T cells^27^. The control group with PBS treatment had an average DC proportion of 1.83%, which increased to 2.4% with AIT01 CS treatment and further increased to 3.34% with AIT01 lysate treatment (Fig. 2A). The population of NK cells, which can directly exhibit anti-tumor effects^27^, also significantly increased in the AIT01 lysate treatment, reaching 3.67%, compared to 0.87% in the control group and 1.36% in the AIT01 CS treatment (Fig. 2B). In addition, the population of γδ T cells, which are generally considered cytotoxic and anti-tumor lymphocytes^28,29^, also significantly increased in the AIT01 lysate treatment, reaching 8.58% (Fig. 2C). Notably, the cytokine production ability of NK cells also significantly increased in the AIT01 lysate treatment. The ability to produce interferon-γ and perforin, associated with the anti-tumor effects of NK cells^27,30^, significantly increased in the AIT01 lysate treatment compared to the control and AIT01 CS groups (Fig. 2D). These findings indicate that AIT01, particularly in its lysate, effectively boosts the population of immune cells and the production of cytokines.

### AIT01 lysate increases the population of CD8^+^ T cells and innate immune cells

Ex vivo studies confirmed that AIT01 lysate stimulates immune cells, but little is known about it in vivo. To address this gap, we investigated whether AIT01 lysate could induce the immune system when administered to mice in vivo. Initially, we identified the most effective method for systemic delivery of AIT01 and concluded that intraperitoneal (i.p.) injection would be most convenient for inducing systemic immune-boosting effects. As a comparison, we used SE28, a *Staphylococcus epidermidis* strain (BGHMC1), which has been previously used for similar purposes^23^.

**Fig. 3:**
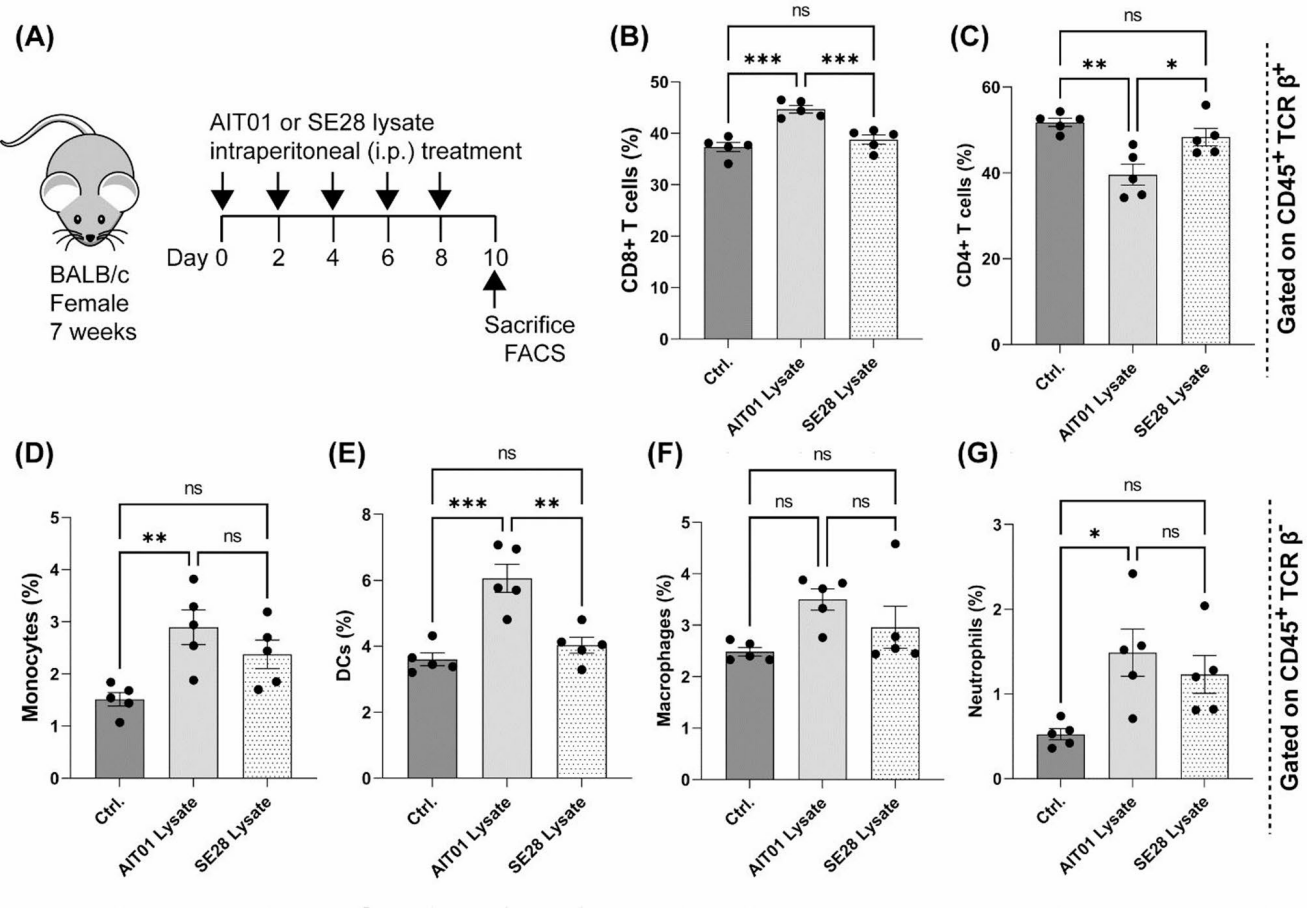
AIT01 lysate elevates the population of CD8^+^ T cells and various types of innate immune cells. The experiment used PBS as the control and SE28 as the bacterial control. (**A**) presents the experimental scheme. AIT01 or SE28 lysate from 10^9^ bacterial cells was intraperitoneally treated in each group (PBS *n* = 5, AIT01 lysate *n* = 5, SE28 lysate *n* = 5). (**B**,**C**) displays changes in the adaptive immune cell populations (CD45^+^ / TCR β^+^ gating). (**D**–**G**) exhibits changes in various types of innate immune cell populations (CD45^+^/TCR β^-^ gating). The asterisks mean: * for a p-value of 0.05, ** for a p-value of ≤ 0.01, and *** for a p-value of ≤ 0.001.

Mice received i.p. injections of AIT01 lysate, SE28 lysate, or PBS at two-day intervals for a total of five times. On the 10th day, immune organs were collected, and flow cytometry analysis was performed (Fig. 3A). The results showed an increase in CD8^+^ T cells in the AIT01 lysate group (44.68%) compared to the PBS (37.34%) and SE28 lysate (38.8%) groups (Fig. 3B). Notably, the CD4^+^ T cell population was decreased by the treatment with AIT01 lysate (Fig. 3C). Various innate immune cells also significantly increased in the AIT01 lysate group, including monocytes (2.89%), dendritic cells (6.06%), macrophages (3.5%), and neutrophils (1.49%), compared to the PBS and SE28 lysate groups (Fig. 3D-G). Although the macrophage distribution did not show statistical significance among the three groups, there was a noticeable trend of increased macrophage proportions in the AIT01 lysate group (3.5%) (Fig. 3F). At the same time, the results for monocytes (Fig. 3D) and neutrophils (Fig. 3G) did not show statistically significant differences between the AIT01 and SE28 groups, although both groups had higher distributions of these cells compared to the PBS control group.

In summary, AIT01 lysate treatment significantly increased the populations of various innate immune cells and CD8^+^ T cells, compared to the control and SE28 lysate groups. This increase in CD8^+^ T cells and dendritic cells highlights the potential of AIT01 lysate as an immunotherapeutic agent, confirming its ability to enhance overall host immunity systemically in vivo.

### Pre-treating AIT01 lysate enhances its anti-tumor effects in a melanoma animal model

**Fig. 4:**
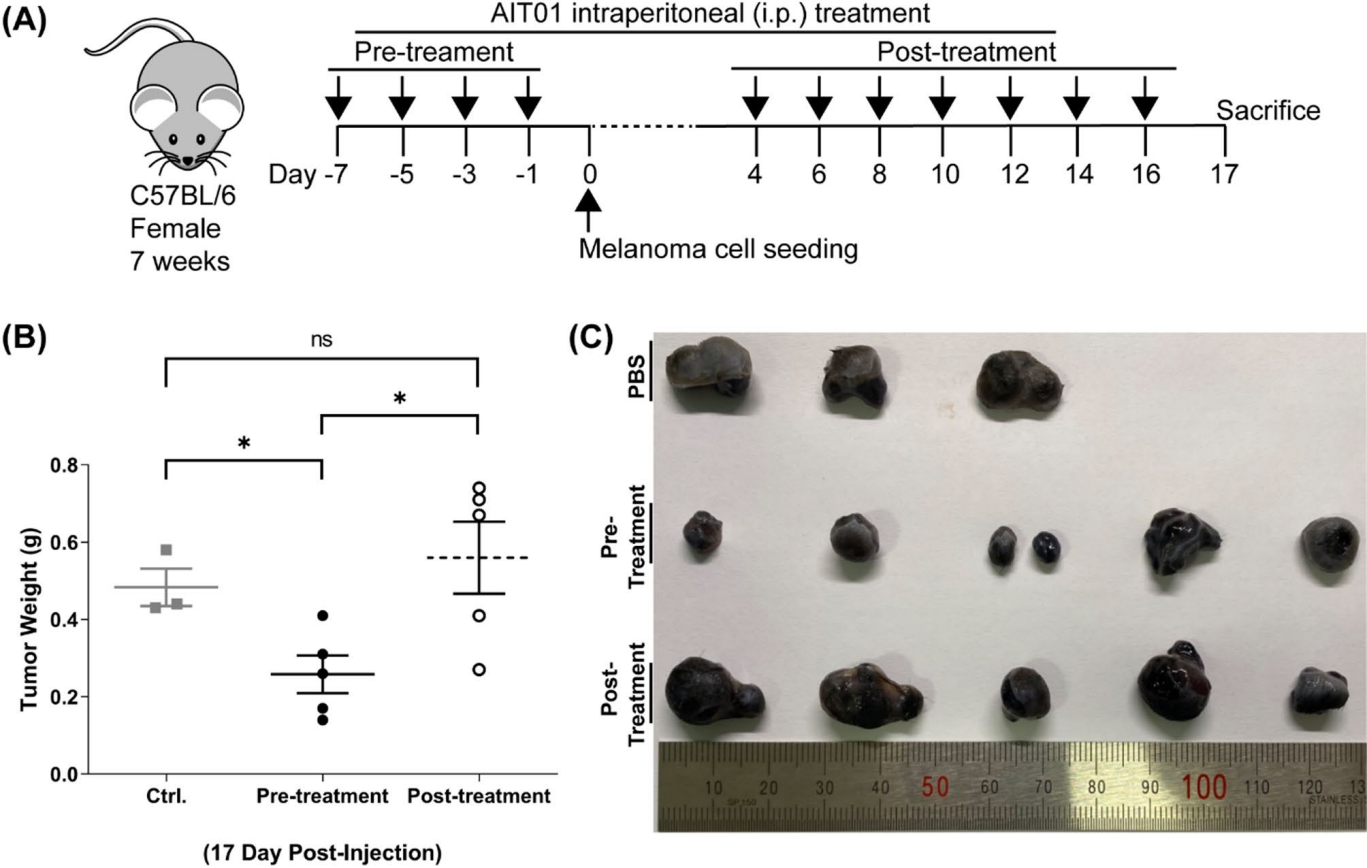
Pre-treatment of AIT01 lysate exhibits anti-tumor effects on a melanoma animal model. (**A**) Depicts the experimental scheme, which was divided into a pre-treatment group that received AIT01 lysate before seeding tumor cells (AIT01 Pre-treatment), and a post-treatment group that received AIT01 lysate after the tumor had developed (AIT01 post-treatment). The lysate collected from 10^9^ bacterial cells was intraperitoneally administered to the mice. The control group was treated with the same volume of PBS as the lysate. We subcutaneously implanted 3 × 10^5^ melanoma cells to induce melanoma in the mice. (**B**) On the 17th day after tumor seeding, mice were sacrificed, and tumor volume and weight were measured. (**C**) A photo of the collected tumor after the sacrifice. The asterisk * denotes a p-value ≤ 0.05.

We conducted an in vivo experiment using a melanoma model to test the hypothesis that AIT01 lysate induces immune system modifications with potential anti-tumor effects. The experiment included three groups: a pre-treatment group, a post-treatment group, and a control group (Fig. 4A). In the pre-treatment group, AIT01 lysate was administered before tumor seeding. This approach was based on previous evidence showing that it boosts the immune system. We hypothesized that administering the lysate before tumor seeding would have a superior inhibitory effect on melanoma compared to post-administration. For the post-treatment group, AIT01 lysate was injected when tumors became visible to the naked eye (Fig. 4A).

All mice were sacrificed on the 17th day after tumor seeding, and their tumors were extracted and measured for volume and weight. The pre-treatment group showed a significantly smaller average tumor weight of 0.25 g compared to 0.48 g in the PBS control group and 0.56 g in the post-treatment group (Fig. 4B). Additionally, Fig. 4C illustrates the noticeable differences in tumor tissue among the groups. These results indicate that pre-treatment with AIT01 lysate significantly inhibits melanoma growth more effectively than post-treatment, supporting its potential as an immunotherapeutic agent.

### The intravenous delivery of AIT01 lysate also effectively suppresses melanoma growth

We reduced the number of melanoma cells to 5 × 10^4^ cells per mouse to lower the initial tumor burden and slow down tumor growth, which helped us to observe the anti-tumor effects of AIT01 lysate more clearly. Unlike the previous experiment, where we used the intraperitoneal (i.p.) route for administering AIT01 lysate, we selected the intravenous (i.v.) route this time to evaluate the effect of a different systemic delivery method.

**Fig. 5:**
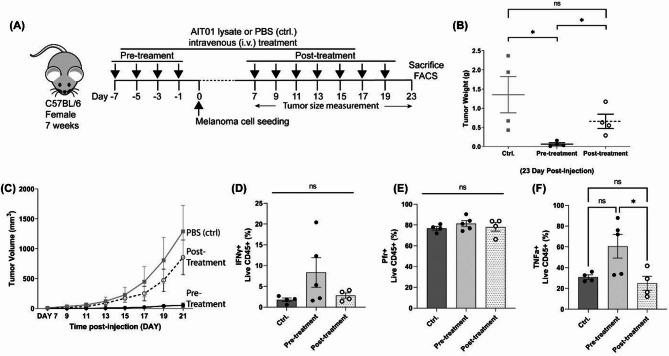
Intravenous delivery of AIT01 lysate also shows anti-tumor effects. (**A**) Illustrates the experimental scheme. The lysate was administered intravenously, and 5 × 10^4^ melanoma cells were subcutaneously seeded on the specified days indicated by arrow symbols. (**B**) The weight of the excised tumor on the 23rd day after tumor seeding. One pre-treatment mouse was considered an outlier according to Grubbs’ test and was removed from the graph. (**C**) The growth curve of the tumor before excision. (**D**–**F**) shows results of flow cytometry analysis of tumor-infiltrated live immune cells (CD45^+^ gating)—groups: PBS (ctrl.) *n* = 4, AIT01 lysate pre-treatment *n* = 5, AIT01 lysate post-treatment *n* = 5. The asterisk symbol indicates a p-value ≤ 0.05.

Approximately seven days after seeding, tumor growth was visually assessed. Both AIT01 lysate pre-treatment and post-treatment groups inhibited melanoma growth compared to the control group (Fig. 5). On the 23rd day after tumor seeding, mice were sacrificed, and tumors were collected and weighed to measure and compare their growth (Fig. 5A). The AIT01 lysate pre-treatment group had an average tumor weight of 0.07 g, the post-treatment group had an average weight of 0.7 g, and the control group had an average weight of 1.4 g (Fig. 5B). Notably, the AIT01 lysate pre-treatment group showed a significantly lower tumor weight than both the AIT01 lysate post-treatment group and the control group, highlighting the superior efficacy of AIT01 lysate pre-treatment (Fig. 5B). Tumor growth curves also showed significant inhibition of melanoma growth in the AIT01 lysate pre-treatment group (Fig. 5C). Furthermore, unlike the previous experiment, the AIT01 lysate post-treatment group also showed a lower average tumor weight than the control group. This outcome could be due to the decreased initial tumor burden resulting from the reduced number of seeded cells, making it easier for the immune system to manage the tumor cells.

Lastly, flow cytometry analysis was performed to observe changes in immune cell populations within the tumor tissue after sacrificing the mice. Given that the B16F10 cell line is non-immunogenic^31^, the population of live immune cells in the tumor tissue was insignificant. Therefore, we directly analyzed the cytokine production ability of the tumor-infiltrating live CD45^+^ population. The results revealed that the AIT01 lysate pre-treatment group had the highest number of immune cells capable of producing anti-tumor cytokines. Specifically, we observed increasing trends in INF-γ (Fig. 5D) and TNF-α (Fig. 5F) levels when AIT01 lysate was pre-treated. However, these differences were not statistically significant due to variability among individual mice. Additionally, no differences in perforin production (Fig. 5E) were detected. Overall, administering AIT01 lysate via the i.v. route showed anti-tumor effects by reducing tumor size but had marginal effects on cytokine production.

### Combination treatment of aPD-1 and AIT01 lysate inhibits melanoma growth significantly more than aPD-1 monotherapy

**Fig. 6:**
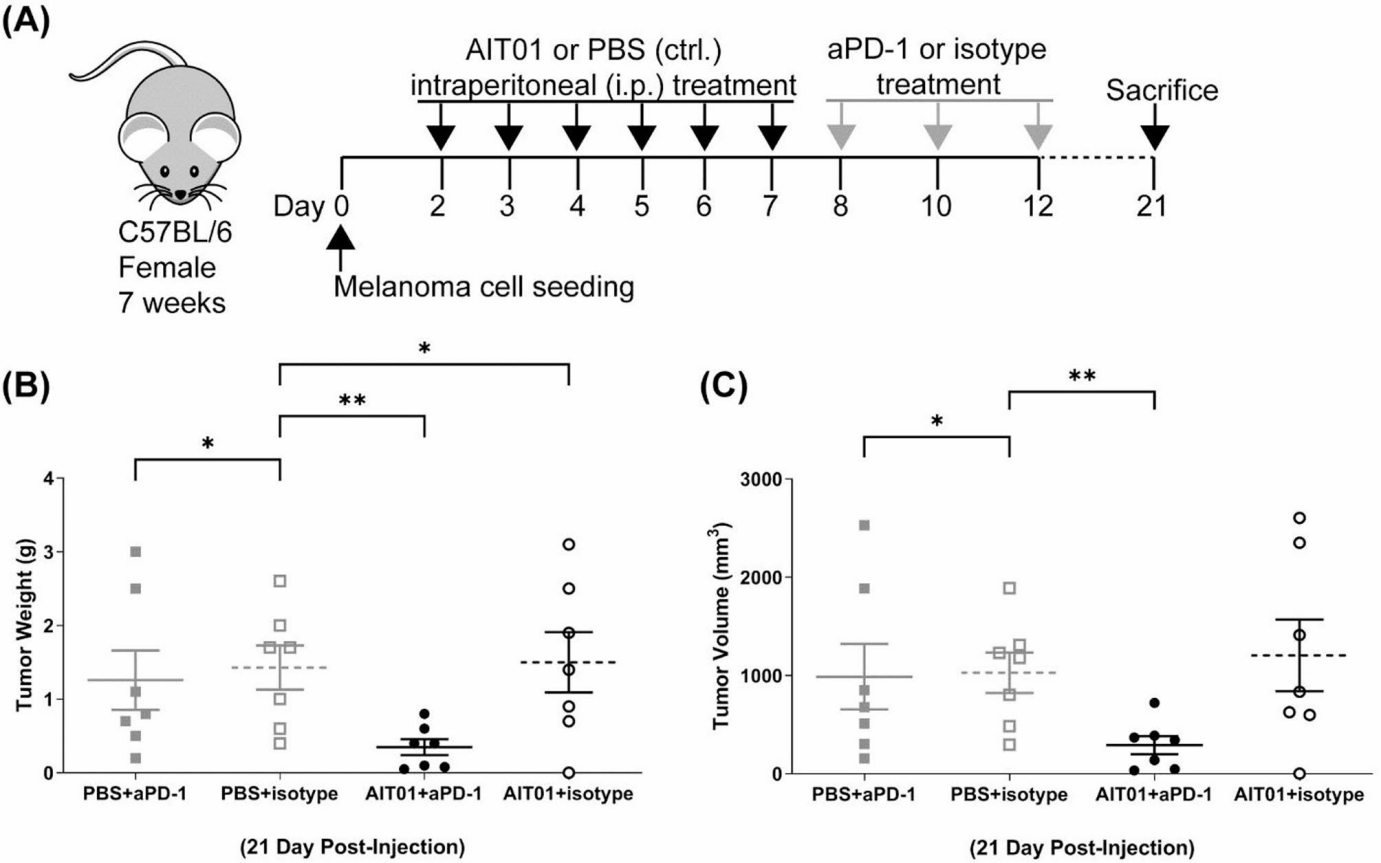
Combination treatment of aPD-1 and AIT01 lysate inhibits melanoma growth more significantly than aPD-1 monotherapy. (**A**) Depicts the experimental design. “PBS + aPD-1” represents the PBS and aPD-1 co-treatment group (*n* = 7), and “PBS + isotype” refers to the PBS and isotype co-treatment group (*n* = 7). “AIT01 + aPD-1” is the AIT01 lysate with the aPD-1 co-treatment group (*n* = 7), and “AIT01 + isotype” is the AIT01 lysate with the isotype co-treatment group (*n* = 7). (**B**,**C**) The weight and volume of the excised tumor on the 21st day after tumor cells inoculation. The asterisks indicate * for a p-value of 0.05 and ** for a p-value of ≤ 0.01.

Finally, we investigated the effect of combination therapy using an anti-PD-1 monoclonal antibody (aPD-1) and AIT01 lysate to maximize melanoma inhibition in the AIT01 lysate post-treatment group. The experimental groups included: aPD-1 monotherapy (PBS + aPD-1), aPD-1 isotype control (PBS + isotype), AIT01 lysate and aPD-1 co-treatment (AIT01 + aPD-1), and AIT01 lysate and isotype control co-treatment (AIT01 + isotype). To evaluate the combined effects of aPD-1 and AIT01 lysate, we minimized the dosage of aPD-1 given to the mice (Fig. S1). Administration of aPD-1 or the isotype control began on day 8 when tumors were visually detectable. Before this, PBS or AIT01 lysate was administered according to the experimental scheme (Fig. 6A). All specimens were administered via the intraperitoneal (i.p.) route.

The results indicated that the AIT01 + aPD-1 group exhibited an average tumor weight of 0.34 g, significantly lower than the AIT01 + isotype group (1.5 g), the PBS + aPD-1 group (1.25 g), and the PBS + isotype control group (1.42 g). Both tumor weight and volume in the AIT01 + aPD-1 group were significantly smaller than in the other groups (Fig. 6B, C). Most tumors treated with PBS + aPD-1 showed a significant reduction in weight and volume compared to the control group. However, a few cases did not exhibit differences, making comparisons challenging in statistical differences. Overall, the combination treatment of aPD-1 and AIT01 lysate demonstrated more significant inhibition of melanoma growth compared to aPD-1 monotherapy, supporting the potential of AIT01 lysate as an effective component in cancer immunotherapy.

## Discussion

This study aimed to evaluate the anti-tumor effects of AIT01 by enhancing the host immune system. Our findings indicate that AIT01, particularly in its lysate form, significantly boosts immune cell viability, proliferation, and functionality, leading to promising anti-tumor outcomes.

The treatment of splenocytes with AIT01 lysate and culture supernatant (CS) demonstrated increased cell viability, with the lysate showing a more pronounced effect (Fig. 1). This indicates that AIT01 lysate is particularly effective in promoting immune cell proliferation, which is essential for robust immune responses against tumors^32,33^. The enhanced immune cell viability suggests that AIT01 lysate can activate immune cells, preparing for effective anti-tumor activity. Flow cytometry analysis revealed significant increases in key immune cell populations, such as NK cells and dendritic cells (DCs) in AIT01 lysate treatment (Fig. 2). NK cells are crucial for their direct tumor-killing capabilities and cytokine production, while DCs are essential for inducing cytotoxic T-cell responses^27,34,35^. These results suggest that AIT01 lysate effectively primes the immune system, enhancing its capacity to mount anti-tumor responses.

Administering AIT01 lysate intraperitoneally to mice resulted in increased populations of CD8^+^ T cells and various innate immune cells, including DCs (Fig. 3). This systemic immune enhancement was associated with significant inhibition of melanoma growth in the pre-treatment group (Fig. 4), likely due to the boosted immune response involving NK cells, DCs, CD8^+^ T cells, and neutrophils. These findings highlight the importance of pre-treatment in enhancing host immunity before tumor establishment for more effective tumor control.

The route of administration influenced the anti-tumor effects of AIT01 lysate. Intravenous administration showed notable tumor inhibition in both pre-treatment and post-treatment groups (Fig. 5). Additionally, combination therapy with AIT01 lysate and anti-PD-1 (aPD-1) monoclonal antibody further enhanced melanoma suppression compared to either treatment alone (Fig. 6). This synergistic effect suggests that AIT01 lysate can augment the effectiveness of existing immunotherapies, providing a more robust anti-tumor immune response.

Recent clinical trials have demonstrated the importance of the gut microbiome in modulating patient responses to anti-PD-1 therapy, with fecal microbiota transplantation (FMT) showing promise in enhancing treatment outcomes for metastatic melanoma patients^36^. Glyceraldehyde-3-phosphate dehydrogenase (GAPDH) and 6-N-hydroxyaminopurine (6-HAP) secreted from *S. epidermidis*, most likely enhance immunity to regulate anti-infective and anticarcinogenic activities, respectively^30,35^.

However, this study has several limitations. While we hypothesized that AIT01 mediates its anti-tumor effects through systemic immune activation, the immune profiling performed was aimed at capturing general trends. Future studies employing more detailed and longitudinal analyses of immune cells, such as dynamics and phenotypic changes following AIT01 administration, will be critical to elucidate its precise mechanism of action, particularly in the pre-treatment group. Moreover, due to experimental constraints, the study was conducted with a limited number of mice. While the pre-treatment group showed consistent anti-tumor effects, increasing the sample size in future studies will be essential to more reliably confirm these results, considering the natural variability in tumor models. Furthermore, research should focus on pinpointing the exact substances in AIT01 lysate that promote immune enhancement, aiming to find compounds that can directly inhibit tumor growth.

In conclusion, our study suggests that a specific type of *S. epidermidis*, AIT01, could serve as a cost-effective and accessible immunotherapeutic agent. By leveraging the immune-stimulatory properties of a skin commensal bacterium, this approach offers a novel and promising strategy for cancer treatment.

## Materials and methods

### Strains and materials

Two strains of *Staphylococcus epidermidis* (SE) were the experimental subject of the study. *S. epidermidis* AIT01 (AIT01) was obtained from the nasal cavity of a healthy donor, while *S. epidermidis* 28 (SE28) was isolated from a patient with chronic allergy symptoms. MACS buffer consists of 5 ml of Fetal Bovine Serum (FBS, Gibco), 4 ml of 0.5 M EDTA (Welgene), and 991 ml of Dulbecco’s phosphate-buffered saline (DPBS, Welgene). T cell media (TCM) was produced by adding Sodium Pyruvate 5 ml (100 mM, Welgene), 1 M HEPES 5 ml (Welgene), MEM Non-essential amino acid 5 ml (100X, Welgene), 2-Mercaptoethanol 0.5 ml (1000X, Gibco), L-Glutamine 5 ml (200 mM, Welgene), Penicillin/Streptomycin 5 ml (Gibco), FBS 50 ml (Gibco), RPMI-1640 Medium 425 ml (Cat. # LM011-01, Welgene). MACS buffer and TCM were stored at 4 °C, and TCM was heated in the water bath at 37 °C right before use.

### Cell culture and sample Preparation

SE was grown on tryptic soy agar (TSA, BD) plate at 37 °C overnight, and a single colony was selected and introduced to 5 ml of tryptic soy broth (TSB, BD). The TSB with a single colony was then incubated overnight in a shaking incubator at 37 °C. Subsequently, we transferred 2 ml of the overnight culture into 200 ml of TSB in a 1 L Erlenmeyer flask. The flask was incubated in a shaking incubator at 37 °C, 230 RPM for 4 h to acquire enough activated bacterial cells. Following the activation, the cells were collected through centrifugation at 4 °C, 4000 RPM for 15 min, and washed twice with phosphate-buffered saline (PBS, Sigma-Aldrich).

For the sample lysate, the bacterial cell pellet was suspended in approximately 10 mL of PBS and autoclaved at 121 °C for 15 min. The lysate concentration was adjusted by adding PBS based on the colony forming units (CFU) value and optical density (OD) at 600 nm of the bacterial lysates. The lysates were stored at -20 °C and thawed immediately before use.

SE supernatant was harvested by culturing SE on a TSA plate at 37 °C overnight, picking a single colony, and introducing it to 10 ml of TSB. The culture was then incubated for 24 h in a shaking incubator set at 37 °C, 230 RPM. Afterward, we precipitated the bacterial cells by centrifugation at 4 °C, 4000 RPM for 15 min. The supernatant above the cell pellet was collected, filtered once using a 0.22 μm syringe filter (Sartorius), and stored at 4 °C.

For anti-tumor experiments, the B16F10 melanoma cell line (Yonsei University College of Medicine) was cultured in Dulbecco’s Modified Eagle’s Medium (DMEM, Welgene) containing 10% FBS (Gibco) and 1% Penicillin/Streptomycin (Gibco). After the fourth passage, we harvested the cells, suspended in DPBS (Welgene), and seeded subcutaneously on the right flank of female C57BL/6 mice in concentrations of 3 × 10^5^ cells / 100 µl, 5 × 10^4^ cells / 100 µl, or 1 × 10^5^ cells/ 100 µl, depending on each experiment set scheme.

### Immune cell viability assay with splenocytes

To obtain splenocytes, a combination of diverse immune cell populations, we sacrificed seven-week-old specific pathogen-free (SPF) BALB/c female mice and acquired spleens. The spleens were then mashed twice using a cell strainer (70 μm, SPL) using MACS buffer and underwent centrifugation to obtain the immune cell pellet. Red blood cells among the cell pellet were lysed using RBC lysis buffer (Biolegend). We suspended the final splenocytes in TCM, counted cell numbers, and adjusted the concentration.

Splenocytes were prepared and added to a 96-well plate (flat bottom, sterile, SPL) at a concentration of 5 × 10^5^ cells per well. SE lysates and supernatants were added to each well with 5, 10, 20, and 40 dilution factors in the same volumes. Subsequently, we incubated the cell cultures in a CO_2_ chamber at 37 °C for 6 and 24 h. The experiments were in triplicate, and cell viability was assessed by adding Cell Counting Kit-8 (CCK-8, Dojindo) 10 µl per every well and OD values, measured by an ELISA reader at 600 nm.

### Ex vivo experiments with splenocytes

Splenocytes suspended in TCM were introduced into a 96-well plate (round bottom, sterile, SPL) with 1 × 10^6^ cells/80 µl concentration. Bacterial lysate or supernatant was treated in a splenocyte mixture. Plates were incubated for 24 h in a CO_2_ incubator at 37 °C, and 4 h before the time endpoint, Golgi stop (BD) was added. Surface staining was performed after the incubation using antibodies described in Tables 1 and 2 and stored for 30 min at 4 °C in the dark. PBS was added to the plates and centrifuged at 4 °C, 2000 RPM for 5 min. Supernatants were removed, and Fixation/Permeabilization solution (Invitrogen) was treated with 100 µl / well and stored at 4 °C for overnights in darkness. The next day, 1x Permeabilization buffer (Invitrogen) diluted by distilled water was added into the wells and centrifuged at 4 °C, 2000 RPM for 5 min. Intracellular staining was performed by antibodies described in Tables 1 and 2 and stored for 30 min at 4 °C in darkness. After the centrifugation at 4 °C, 2000 RPM for 5 min, the supernatants were removed. Cell pellets were resuspended in PBS and analyzed by a SONY ID7000 flow cytometer (Sony).

### In vivo experiments with mice

Mice used in this study were C57BL/6 and BALB/c female, seven weeks old, and specific pathogen-free (SPF) mice (Orient Bio). All methods were performed in accordance with the animal experimental guidelines and regulations approved by the Department of Animal Resources of Yonsei Biomedical Research Institute. Additionally, we adhered to the ARRIVE guidelines, and all data were reported in compliance with these standards. To euthanize the mice, we first administered CO_2_ asphyxiation, followed by cervical dislocation to confirm death. Following a period of acclimatization, the mice were exploited in the experiment. We implemented AIT01 and SE28 lysate single-treatment experiments to figure out specific changes that happened to immune cells with the lysate administration in vivo. For the experiments, we injected lysates into the peritoneal cavity of mice. The injection dose was 10^9^ CFU / 100 µl per mouse, and injections were given five times at a 2-day interval. On the 10th day after the start of administration, spleens were extracted, and flow cytometry analysis was performed.

Tumor volume was measured every two days, and substances, such as AIT01 lysate, PBS, aPD-1, and isotype control, were injected intraperitoneal or intravenously according to each experiment’s scheme. We described specific schemes and injection routes in each Figure. Every i.p. or i.v. injection volume was 100 µl / mouse. aPD-1 (Cat. # BE0146, BioXcell) and its isotype control (Cat. # BE0089, BioXcell) were used for aPD-1 and AIT01 lysate combination treatment experiment set, stored at 4 °C. After the sacrifice, we obtained tumors and measured their volumes and weights. Subsequently, the obtained immune organs and tumors were analyzed using flow cytometry. We primarily sacrificed any individual whose tumor size exceeded 2 cm during the experiment due to ethical considerations.

The tumor tissues obtained were cut into fine pieces and poured into the digestion solution (pre-warmed 48.8 ml of DMEM (Welgene) with Collagenase type IV (Gibco), 100x DNase I (Sigma-Aldrich), and FBS (Gibco). The mixture was placed in a shaking incubator for 30 min at 37 °C, 240 RPM. Tumor tissues with digestion solution were mashed twice by a cell strainer (70 μm, SPL), and the digestion reaction was stopped by adding MACS buffer. After the centrifugation at 4 °C, 2000 RPM for 5 min, only the cell pellets were suspended in TCM for use. Subsequently, each single-cell suspension from the tumors and spleens was inoculated into a 96-well plate (round bottom, sterile, SPL). Next, only the cytokine panel was added to a stimulation cocktail (500x, Thermo Fisher Scientific) with CD107a and incubated in a CO_2_ incubator for 4 h at 37 °C. The cells in the 96-well plates were stained using antibodies listed in Tables 1 and 2. Fixation/Permeabilization solution (Invitrogen) and 1x Permeabilization buffer (Invitrogen) were used in the same manner as in the ex vivo experiment above. Finally, cell pellets were resuspended in PBS, analyzed by a SONY ID7000 flow cytometer (Sony), and analyzed by FlowJo (BD).

### Flow cytometry analysis

We divided flow cytometry panels into two, considering the impact of the cell stimulation cocktail on immune cell populations. One panel was set for checking immune cell population changes, and the other was for checking immune cells’ cytokine production ability changes. For the population panel, staining monoclonal antibodies are described in Table 1, and for the cytokine panel, antibodies are described in Table 2.

**Table 1:** Panel for identifying changes in immune cell populations.

Antibody name	Fluorophore	Clone name	Source
CD11c	Pacific Blue (PB)	N418	Biolegend
Live/Dead	Zombie Aqua	–	Biolegend
TCR *γδ* or Ly6C	Brilliant Violet 605 (BV605)	GL3 or HK1.4	Biolegend
TCR β	Brilliant Violet 650 (BV650)	H57-597	Biolegend
CD4	Brilliant Violet 785 (BV785)	GK1.5	Biolegend
Ly6G	Fluorescein isothiocyanate (FITC)	RB6-8C5	Biolegend
NK 1.1	Phycoerythrin (PE)	PK136	Biolegend
CD11b	Peridinin chlorophyll protein-Cyanine5.5 (PerCP-Cy5.5)	M1/70	Biolegend
F4/80	PE-Cyanine7 (PE-Cy7)	BM8	Biolegend
Foxp3	Allophycocyanin (APC)	FJK-16s	eBioscience
CD45	Allophycocyanin-R700 (APC-R700)	30-F11	BD
CD8	Allophycocyanin- Cyanine7 (APC-Cy7)	53 − 6.7	Biolegend

**Table 2:** Panel for identifying changes in cytokine production ability of immune cell populations.

Antibody name	Fluorophore	Clone name	Source
IL-5	Brilliant Violet 421 (BV421)	TRFK5	Biolegend
Live/Dead	Zombie Aqua	–	Biolegend
TCR *γδ*	Brilliant Violet 605 (BV605)	GL3	Biolegend
TCR β	Brilliant Violet 650 (BV650)	H57-597	Biolegend
CD4	Brilliant Violet 785 (BV785)	GK1.5	Biolegend
TNF-α	Fluorescein isothiocyanate (FITC)	MP6-XT22	Biolegend
NK 1.1	Phycoerythrin (PE)	PK136	Biolegend
Interferon-*γ*	Peridinin chlorophyll protein-Cyanine5.5 (PerCP-Cy5.5)	XMG1.2	Biolegend
CD107a	PE-Cyanine7 (PE-Cy7)	1D4B	Biolegend
Perforin	Allophycocyanin (APC)	S16009A	Biolegend
CD45	Allophycocyanin-R700 (APC-R700)	30-F11	BD
CD8	Allophycocyanin- Cyanine7 (APC-Cy7)	53 − 6.7	Biolegend

### Statistical analysis

Data are expressed as mean ± standard error of the mean (SEM). Statistical analysis of data was performed via graphs and GraphPad Prism software (ver. 9.4.1, GraphPad) by One-way ANOVA test and t-test to determine the significance of data. P-values < 0.05 were considered to be statistically significant.

## Supplementary Information

Below is the link to the electronic supplementary material.

Supplementary Material 1

## Data Availability

All data generated or analysed during this study are included in this article.
