# Supplementary material for: Enhancing melanoma treatment through systemic delivery of an immune boosting Staphylococcus epidermidis strain
Source: Sci Rep. 2025 Oct 21;15:36697. doi: 10.1038/s41598-025-20581-x (PMC12540718; doi:10.1038/s41598-025-20581-x)
Supplement: Supplementary file 1 — Supplementary Material 1 [file 41598_2025_20581_MOESM1_ESM.docx]

**Figure S1. Anti-cancer effects of aPD-1 on melanoma tumor growth.** 100 µg / 200 µg of aPD-1 or its isotype was administered to assess whether aPD-1 effectively inhibits the progression of melanoma. 1 x 10^5^ melanoma cells were seeded on day 0 to induce skin cancer. This figure illustrates changes in melanoma tumor volume and weight on specific days when treated with aPD-1 or the isotype. The asterisk symbol shows a p-value ≤ 0.05.
